# A clarion call to the community of current and potential journal reviewers

**DOI:** 10.1186/s12967-018-1574-8

**Published:** 2018-07-19

**Authors:** Michael N. Liebman, Franco Marincola

**Affiliations:** 1 IPQ Analytics, LLC, Kennett Square, USA; 2 Refuge Biotechnologies, Menlo Park, CA 94025 USA

If you are reading this commentary then it is most probable that you have been invited and even participated in the review of submissions to a scientific journal at some point in your career. For those of you who have performed reviews, thank you for your contribution to the advancement of high quality science. The purpose of this commentary is to encourage and recruit additional reviewers as this is the critical fuel that makes a journal successful. As journal or section editors, we recognize that reviewers are perhaps more critical than authors and much more difficult to recruit.

Scientists sometimes forget that they exist within a closed cycle system. They are funded to carry out research, which they must publish, to hopefully obtain tenure and additional funding with which they are funded to carry out research….and while most scientists see reviewing grant proposals as a duty and honor and rarely refuse, many neglect to see reviewing journal submissions in the same light. Without a continuous stream of reviewers and reviews, your submission can be significantly delayed in the first step of write–revise–publish, and negatively impact tenure and promotion decisions and supporting material for grant submissions. Perhaps this has even contributed to the incredible increase in the number of predatory journals because of the ability to exchange paying unwarranted page charges for rapid, but poorly regulated publication.

Let’s examine the most common reasons that editors receive for refusing an invitation to review a journal submission, but first, the invitation:

“I am writing to invite you to serve to review the manuscript: “*SOX 2, a new genomic marker in patients presenting with (ICD10) S10.87XA where BRCA1 status provides guidance for treatment*?” (contrafaco)…

1. “It’s not my area of expertise”….

The likelihood that you are an expert in this work (NB: S10.87XA is a condition known commonly as “hickey”) is incredibly small (p ≪ 0.00000001) but, as a scientist, you can objectively review many components of the study, e.g. methods, data, analysis, and also state what you specifically have or have not reviewed. This can still be very valuable to both the editor and the author in evaluating and improving the manuscript and the research. In addition, if structured appropriately, the opportunity exists for you to also gain insights into methodologies and diseases, etc. that could prove useful in expanding your own research perspective.

*Potential solution* There should be a “checklist” provided to and completed by the author upon submission that goes beyond the current general classification being used, and that provides more detail about the methodologies and/or analytics, data source/collection, disease/condition being studied, public/private healthcare/population environment, etc. A similar checklist, to be completed by potential reviewers, and that should definitely include any and all authors who have published in that journal, could be used to better evaluate and match manuscript to reviewer. General categorizations, e.g. “cardiovascular”, do not adequately address this issue.

2. “I am busy…I don’t have time”

Of course you are busy…. A successful career in science has become increasingly challenging because of funding issues and coopertition between academia and industry, especially academic spin-out companies. And some potential reviewers sit in more than one world at the same time. The scientific community, however, exists as a somewhat closed group defined by essential requirements of both advanced training and experience. We all need to remember that this system maintains a form of symmetry…if everyone is to busy working and writing papers (or grants) to review a limited number of manuscripts, then we should expect that our submissions also will meet reviewers who “are too busy”. It is perhaps appropriate to consider the responsibility to perform reviews as a “professional tax”…and it is also appropriate to expect not to be over-taxed.

*Potential solution* Journal editors can “assign” credits for performing reviews, with the total points being stored in the reviewer’s “account” and maintained by an honest broker i.e. an academic institution or non-profit organization/professional society, across the scientific community. Reviewers could be appropriately acknowledged, annually, for their contributed activity and possibly, some publishers might offer reduced publication fee opportunities. This documentation could/should be further considered in academic promotion and review procedures.

*And if you are “on vacation” or “travel”, it is also appropriate to suggest when you might be able to return the review, even if after the proposed date, so that the editor can respond accordingly.*

3. ‘Invited but no response”

Unfortunately, this category is larger than can be justified and the total lack of response is inappropriate and unprofessional. An editor appreciates having a response and commitment, either to review or not to review. And editors and publishing systems do keep track of their reviewer pools. This is a list where you likely may not wish to be included.

We welcome your response and thoughts…and even more importantly, your potential interest in joining the group of active reviewers who help to advance the science in a responsible and quality manner.

